# Impact of molecular diagnostic techniques on the acute respiratory infection sentinel surveillance program, Antioquia, Colombia, 2022

**DOI:** 10.3389/fepid.2024.1519378

**Published:** 2025-01-10

**Authors:** María Angélica Maya, Celeny Ortiz, Francisco Averhoff, Mabel Carabali, Laura S. Pérez-Restrepo, Karl Ciuoderis-Aponte, Ana Isabel Davila, Diego Bastidas, Seti Buitrago, Gavin A. Cloherty, Michael G. Berg, Alan Landy, Juan P. Hernandez-Ortiz, Paulina A. Rebolledo, Jorge E. Osorio

**Affiliations:** ^1^ GHI One Health Colombia, Universidad Nacional de Colombia, Medellín, Colombia; ^2^ Departmental Health Secretariat of Antioquia, Medellín, Colombia; ^3^ Infectious Diseases Research, Abbott Diagnostics, Abbott Park, IL, United States; ^4^ Infectious Disease Research Abbott and the Abbott Pandemic Defense Coalition (APDC), Chicago, IL, United States; ^5^ Department of Epidemiology, Biostatistics, and Occupational Health, McGill University, Montreal, QC, Canada; ^6^ Control and Prevention Infection, San Vicente Fundacion Hospital, Medellín, Colombia; ^7^ Pediatrics, San Vicente Fundacion Hospital, Medellín, Colombia; ^8^ Virology and Molecular, Public Health Laboratory of Antioquia, Medellín, Colombia; ^9^ Department of Medicine and Department of Immunology Microbiology, University of Texas Medical Branch, Galveston, TX, United States; ^10^ Division of Infectious Diseases, Department of Medicine and Global Health, Emory University School of Medicine and Rollins School of Public Health, Atlanta, GA, United States; ^11^ Global Health Institute, University of Wisconsin-Madison, Madison, WI, United States

**Keywords:** pathogens, acute respiratory infection, immunofluorescence, molecular methods, multiplex polymerase chain reaction (mPCR), epidemiology

## Abstract

**Objectives:**

Surveillance of acute respiratory infection (ARI) informs vaccination, preventive, and management decisions. In many countries, immunofluorescence is the cornerstone for ARI surveillance. We aimed to determine the effect of adding multiplex polymerase chain reaction (mPCR) to conventional surveillance in ARI.

**Methods:**

Respiratory samples from patients with influenza-like illness (ILI) and severe acute respiratory infection (SARI) were tested by a conventional approach [direct immunofluorescence (DIF) and SARS-CoV-2 PCR, and a subset of samples underwent routine testing]. Negative specimens were tested by multiplex PCR (mPCR), and remain negative samples were sequenced. Descriptive, multivariable regression analyses were conducted.

**Results:**

Between March and June 2022, 299 patients were enrolled. Pathogens were detected in 43.8% of samples (131/299) tested by the conventional approach. Of the 168 negatives after the conventional approach, 157 (93.4%) were positive by mPCR, increasing the detection rate to 96.3% (288/299). With the conventional approach, the most frequent pathogen was respiratory syncytial virus (50.3%, 66/131), whereas with mPCR it was *Haemophilus influenzae* (37.5%, 63/168). mPCR significantly improved pathogen detection in ARI surveillance (Adjusted Incidence Rate Ratios 4.22 95% IC 4.22–5.85).

**Conclusion:**

Adding mPCR to respiratory surveillance conventionally based on DIF significantly enhanced virus and bacteria detection. mPCR should be considered for routine ARI surveillance.

## 1 Introduction

Severe acute respiratory infections (SARI) were estimated to be the fourth leading cause of death worldwide in 2019, before the SARS-CoV-2 pandemic (1). Although the burden had decreased over the prior decade, SARI accounts for a significant number of hospitalizations, deaths, and disability-adjusted life years (DALYs) lost (2). Identifying the pathogens responsible for SARI is the first step to identify at-risk populations, and implement appropriate prevention and treatment strategies. However, in resource-limited settings technological resources and financial limitations impact the selection of diagnostic tools and surveillance algorithms.

The Pan American Health Organization (PAHO) recommends using antigen detection, immunofluorescence, ELISA, pathogen isolation, or polymerase chain reaction (PCR) to identify pathogens associated with respiratory infections (2). The PCR assay, in particular, is highly recommended for detecting influenza viruses. In Latin America, immunofluorescence techniques are frequently used to surveillance common respiratory viruses, and PCR for the detection of influenza virus and SARS-CoV-2 has been increasingly implemented in the region (3–5).

The National Health Institute leads a sentinel surveillance program for acute respiratory infection (ARI) in Colombia. This program relies on a national network of health institutions and diagnostic laboratories. The surveillance network in the Department of Antioquia is based on three sentinel hospitals that provide samples from patients with influenza-like illness (ILI) and severe acute respiratory infection (SARI). Identification of pathogens in ILI and SARI cases is mainly obtained through immunofluorescence, with selected samples undergoing additional testing with PCR for influenza and SARS-CoV-2, and bacterial culture. With this approach, the National IRA surveillance system reported at the end of 2023 that respiratory syncytial virus (RSV) was the most frequently detected pathogen (25.5%), followed by rhinovirus (19%), and in the third place adenovirus, parainfluenza and SARS-Co-2 (each one 10%) (6).

The low sensitivity of immunofluorescence assays and routine bacteria cultures results in a sizable proportion of samples being negative, limiting the impact and outcome of an active surveillance program for respiratory pathogens (7, 8). Molecular techniques such as multiplex polymerase chain reaction (mPCR) and sequencing allow for the simultaneous detection of several respiratory pathogen targets with higher sensitivity than immunofluorescence (9, 10). These molecular techniques are rarely used for testing ARI in Colombia and are not routinely used in the national surveillance program. Studies are needed to inform policies regarding implementing more advanced diagnostic approaches to conducting ARI surveillance in low-income countries. This study aimed to assess the impact of mPCR testing and sequencing on samples that test negative from patients presenting with SARI and ILI after testing with the conventional approach that includes immunofluorescence and SARS-CoV-2 PCR for all the samples and some additional testing according to physicians' criteria.

## 2 Materials and methods

### 2.1 Patient selection and sampling

Patients of any age seeking medical attention in the emergency room with ILI and SARI were recruited from March 2022 to June 2022 from three sentinel sites in the Department of Antioquia (Colombia), including San Vicente Foundation Hospital in Medellín (SVFH), Hospital San Juan de Dios in Yarumal, and Hospital San Rafael in Yolombó. Patients with signs and symptoms that met the case definition for ILI and SARI were asked to participate in this study. If they met the case definition and agreed to participate, they were consented and enrolled at the time of hospitalization. SARI was defined as a person who presented with a cough accompanied by tachypnea, fever, or history of fever (body temperature above 38°C), with onset of symptoms within the last 10 days, and requiring hospitalization. The case definition of ILI was the same as SARI, except the patient did not require hospitalization.

### 2.2 Clinical data and sample collection

After informed consent was obtained, one nasopharyngeal wash or respiratory swab was obtained from each consenting participant. Sample collection followed standard respiratory specimen collection practices. Samples were kept at 4°C until storage at −80°C.

Demographic and clinical data from enrolled patients were obtained from their electronic medical records. Microbiology results from provider-ordered standard-of-care testing were obtained from the laboratory information system. The results of the mPCR were not available to the treating physicians.

### 2.3 Laboratory testing

For this study, the conventional approach was defined as a combination of Direct immunofluorescence (DIF), SARS-CoV-2 PCR, and other routine diagnostic tests performed per standard of clinical care (bacteria cultures, Mycoplasma serology, BioFire ® Filmarray ® Pneumoniae panel, Mycobacterium tuberculosis PCR). Direct immunofluorescence assay included detection for Respiratory syncytial virus (RSV), adenovirus (ADV), influenza A virus (Flu A), influenza B virus (Flu B), parainfluenza virus types 1–3 (PIV1-3), and metapneumovirus (hMPV), and was performed using a D3 Ultra direct fluorescent antibody Respiratory Virus Screening identification Kit (Diagnostic Hybrids Inc., USA) following the manufactureŕs instructions. Samples that tested positive for influenza virus were subtyped using the CDC protocol (11). Nucleic acids were extracted using the Genolution NX-48S viral NA kit from Korea at the Antioquia Public Health Laboratory. All samples were assayed for SARS-CoV-2 by PCR (Genefinder COVID-19 Plus realamp kit, Onsang, Korea) following the manufacturer's instructions.

Respiratory samples that remained negative after the conventional approach were submitted to the One Health Genomic Laboratory at the Universidad Nacional de Colombia in Medellin for mPCR testing and sequencing. Total nucleic acids (DNA and RNA) were extracted using MagMAXTM Viral/Pathogen kit for the KingFisher system (Thermo Scientific, USA) following the manufacturer's instructions. Then, mPCR was conducted using four Allplex respiratory panels assays (Seegene Inc, Korea) for the detection of influenza A, A-H1, A-H1pdm09, A-H3, B; RSV A, RSV B, ADV, Enterovirus, hMPV, PIV1-4; Bocavirus 1/2/3/4, Coronavirus 229E, NL63, and OC43, *Bordetella pertussis*, *Bordetella parapertussis, Chlamydophila pneumoniae, Mycoplasma pneumoniae, Legionella pneumophila, Streptococcus pneumoniae*, and *Haemophilus influenzae* with the CFX96 Touch Real-Time PCR Detection System (Bio-Rad, California, USA). All testing was performed according to the manufacturer's instructions. Data obtained were analyzed using Seegene Viewer software (https://www.seegene.com/software/seegene_viewer).

Negative samples after mPCR testing were sequenced using *Respiratory Pathogen ID/AMR Enrichment* Kit (Illumina, Inc., San Diego, CA) for detection of 180+bacteria, 40+viruses, and 50+fungi (https://www.illumina.com/products/by-type/sequencing-kits/library-prep-kits/respiratory-pathogen-id-panel.html). Sequencing was performed on the Illumina Miseq instrument (Illumina, USA). A 300-bp paired-end read sequencing (2 × 150) with expected depths of 2 million reads per sample was targeted. Analysis of sequencing data was accomplished using the automated Explify Respiratory Pathogen ID/AMR Panel (RPIP)—Data Analysis Solution (v2.1.2; IDbyDNA) accessed via Illumina BaseSpace. The sequences obtained were submitted to the SRA database.

### 2.4 Data analysis

Double data entry was performed using a Microsoft Excel spreadsheet (Excel 2010 (14.0). Patients undergoing the conventional approach were categorized into those with positive and negative results. Groups were compared based on location, age, past medical history, signs and symptoms, diagnosis type, treatment, and testing diagnostic results. Continuous variables were expressed as median and interquartile range (IQR). Categorical variables were expressed as proportions.

Multivariable Poisson Regression analysis was used to model the number of microorganisms per sample using the mPCR for positive samples with the conventional approach and negative samples with that approach simultaneously. An exploratory analysis was done with symptoms (odynophagia, rhinorrhea, conjunctivitis, headache, breathiness, diarrhea), comorbidities [asthma, Chronic Obstructive Pulmonary Disease (COPD), diabetes, HIV, heart disease, neoplasia, malnutrition, obesity, kidney disease, smoking, hypertension], chest radiographic findings, current diagnosis (asthma crisis, bronchiolitis, COPD decompensation, pneumonia, rhinopharyngitis, sinusitis, tracheitis), time to medical attention, time to hospitalization. These covariables were adjusted by age and sex. Also, a Bayesian Hierarchical Poisson Regression analysis was used to estimate the number of samples with microorganisms identified using the mPCR test as a function of the selected predictors, accounting for the temporal trend with a random intercept for the week, considering the conventional approach and including mPCR. Analyses were conducted using R version 4.03 (R Foundation for Statistical Computing).

### 2.5 Ethical considerations

Written informed consent was obtained from all participants. For patients aged 17 and younger, informed assent was obtained in addition to the parents’ or legal guardians' consent. Samples and questionnaire data were anonymized before analysis. The study protocol was reviewed and approved by the Ethics committee of the San Vicente Foundation Hospital (number 02-2022).

## 3 Results

Between March and June 2022, 360 patients were tested with the conventional approach. We excluded 61 patients; 17 did not meet the study criteria, 2 had limited access to their clinical records, one was enrolled twice while visiting two different hospitals on the same day, and 41 had insufficient sample volume to test with mPCR. Of the 299 patients enrolled, the median interval between the onset of symptoms and the consultation date was 2 days (IQR 1–4 days). Patients over 20 years old were minimal, with only 20 individuals (6.6%) falling into this category. Hospitalization was required for 92.9% (278/299) of the patients, and nearly half of the patients resided in urban areas, 90.3% (270/299) (Supplementary Figure S1).

**Figure 1:**
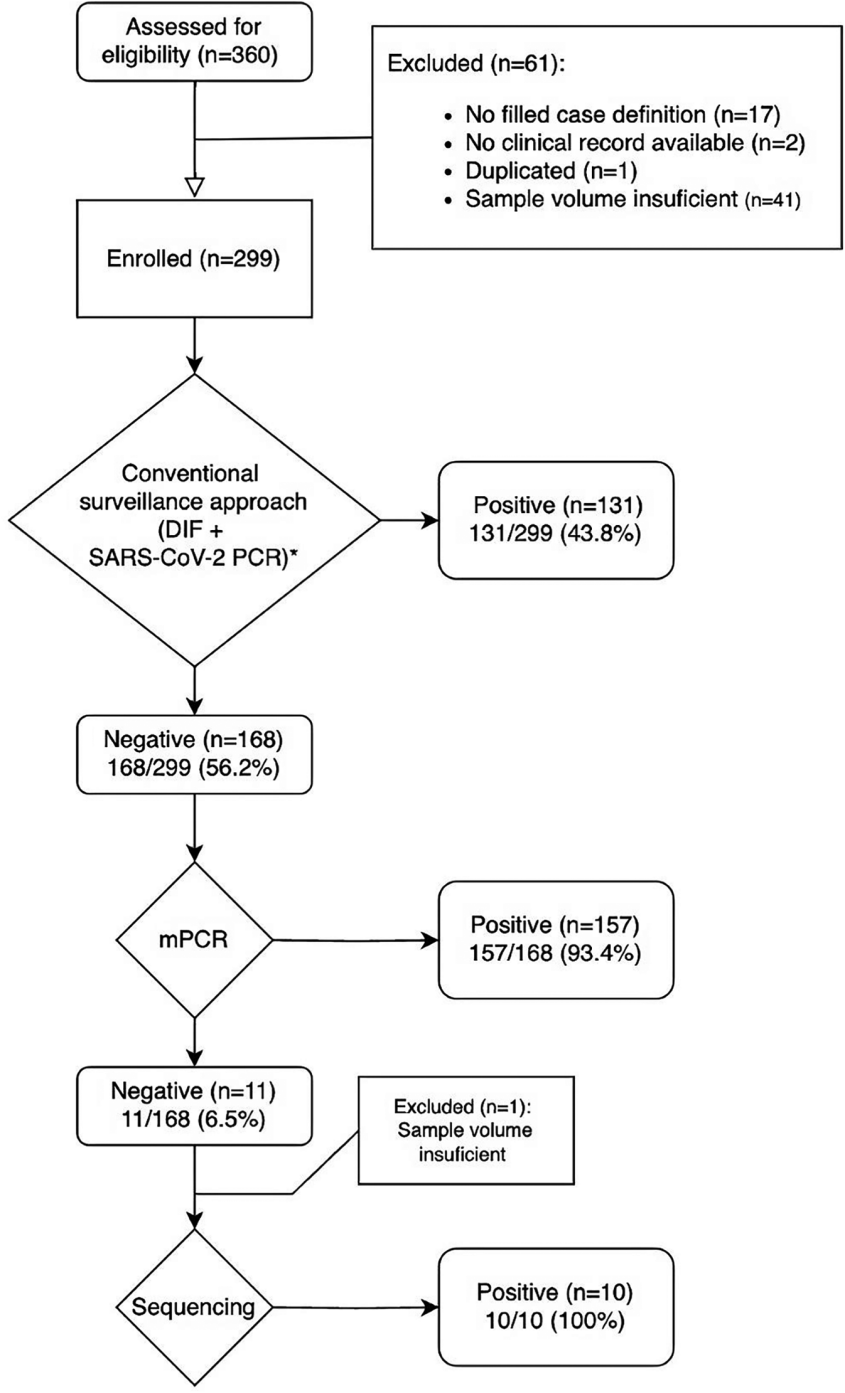
Flow chart of the number of samples recruited and tests performed. DIF, direct immunofluorescence. *All the samples were analyzed by DIF and SARS-CoV-2 PCR. Other tests were ordered for physicians according to their criteria: bacteria cultures, mycoplasma serology, BioFire® Filmarray® pneumoniae panel, mycobacterium tuberculosis PCR in 41 patients.

Overall, the conventional approach, which included DIF, SARS-CoV-2 PCR, and additional testing order by the attending clinician (43/299; 14.3%), resulted in 131/299 (43.8%) with at least one pathogen identified (Figure 1). Testing with DIF yielded a positivity rate of 32.7% (98/299), while tests for SARS-CoV-2 PCR were positive among 9.36% of the specimens tested (28/299). Of those 43 samples, which were tested with complementary assays, 19 patients had a positive test result (8 by BioFire, one via serology for Mycoplasma, one by Mycobacterium tuberculosis PCR, and nine by routine bacterial cultures from various sites (5 by tracheal aspiration, two by blood cultures, and two by pleural fluid culture).

**Figure 2:**
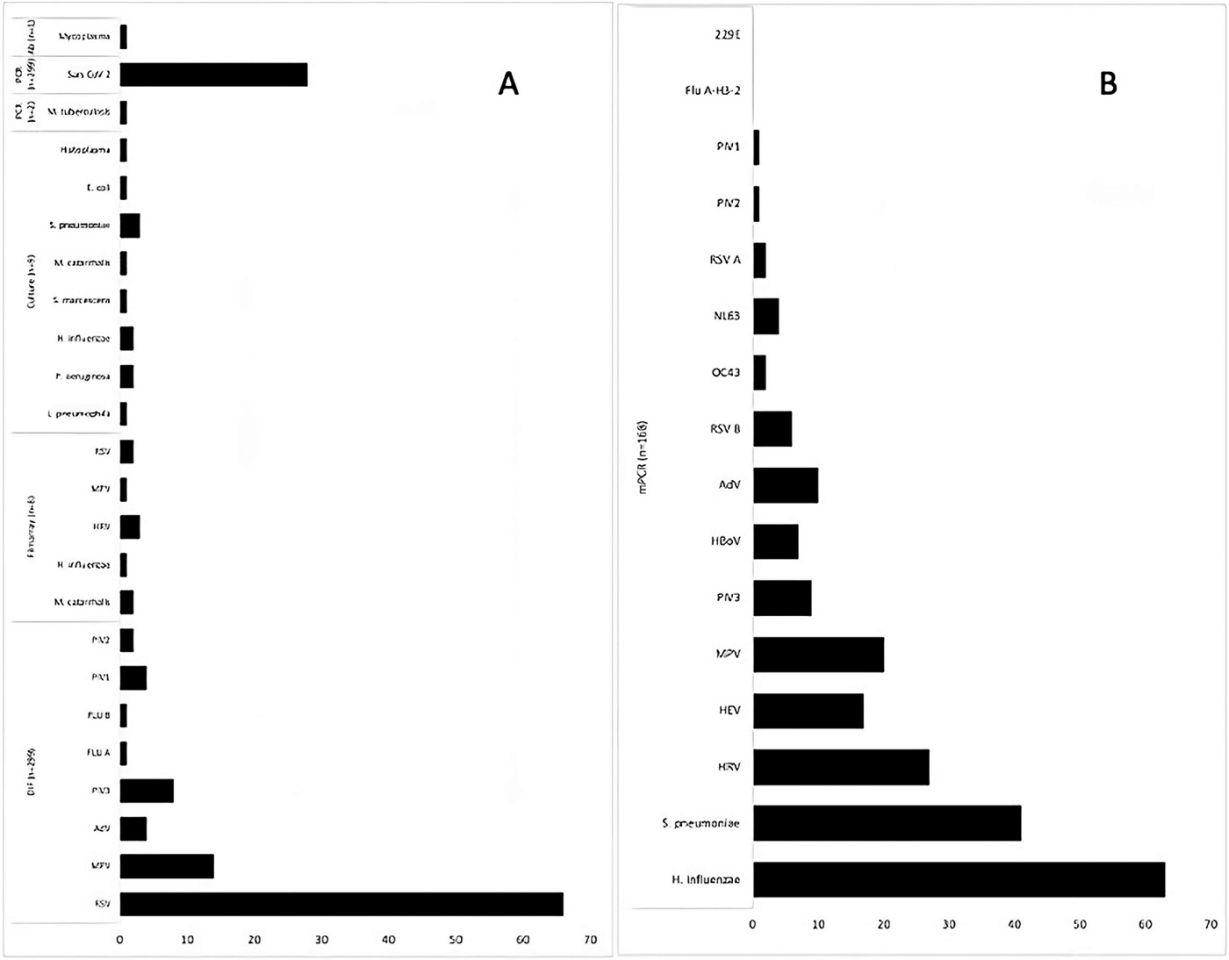
The frequency of the microorganisms is identified by each assay. **(A)** Show the assays used in the conventional approach. **(B)** Show microorganisms identified by mPCR applied to negative samples after the conventional approach. The number of samples per assay is shown in parentheses. RSV, respiratory syncytial virus; RSV A, respiratory syncytial virus A, RSV B, respiratory syncytial virus B; MPV, metapneumovirus; AdV, adenovirus; PIV3, parainfluenza virus 3; PIV1, parainfluenza virus 1; FLU A, influenza A; FLU B, influenza B; HRV, rhinovirus; NL63, coronavirus NL63; OC43, coronavirus OC43; 229E, coronavirus 229E; HEV, enterovirus; HboV, bocavirus.

The most commonly identified pathogens detected by the conventional approach were RSV (50.3%, 66/131), SARS-CoV-2 (21.3%, 28/131), hMPV (10.6%, 14/131), and PIV 3 (6.1%, 8/131) (Figure 2). Of the 66 patients with RSV, 50.0% (33/66) were children under one year of age, and of the 28 with SARS-CoV-2, 39% (11/28) were children between 2 and 4 years old. Co-infections were detected among 16% (21/131), the most frequent co-detection were RSV and SARS-CoV2 (28.5%, 6/21), RSV with any other virus (23.8%, 5/21), and SARS-CoV-2 with any virus other than RSV (19.0%, 4/21).

After testing with the conventional approach, 168 samples (56.2%) remained negative, of those, Allplex ® mPCR was performed. A single pathogen was detected in 16% (27/168), two in 20.2% (34/168), and three or more in 57.1% (96/168). The overall yield by mPCR testing was a positivity rate of 93.4% (157/168). The most frequently identified pathogens were *H. influenzae* (37.5%, 63/168), *S. pneumoniae* (24.4%, 41/168), Rhinovirus (16.1%, 27/168), Metapneumovirus (11.9%, 20/168), and Enterovirus (10.1%, 17/168) (Figure 2). Furthermore, the most frequent co-detections were *H. influenzae* and *Rhinovirus* (6.9%, 9/130), followed by *H. influenzae* and *Enterovirus* (3.8%, 5/130). The conventional approach detected none of these pathogens.

**Figure 3:**
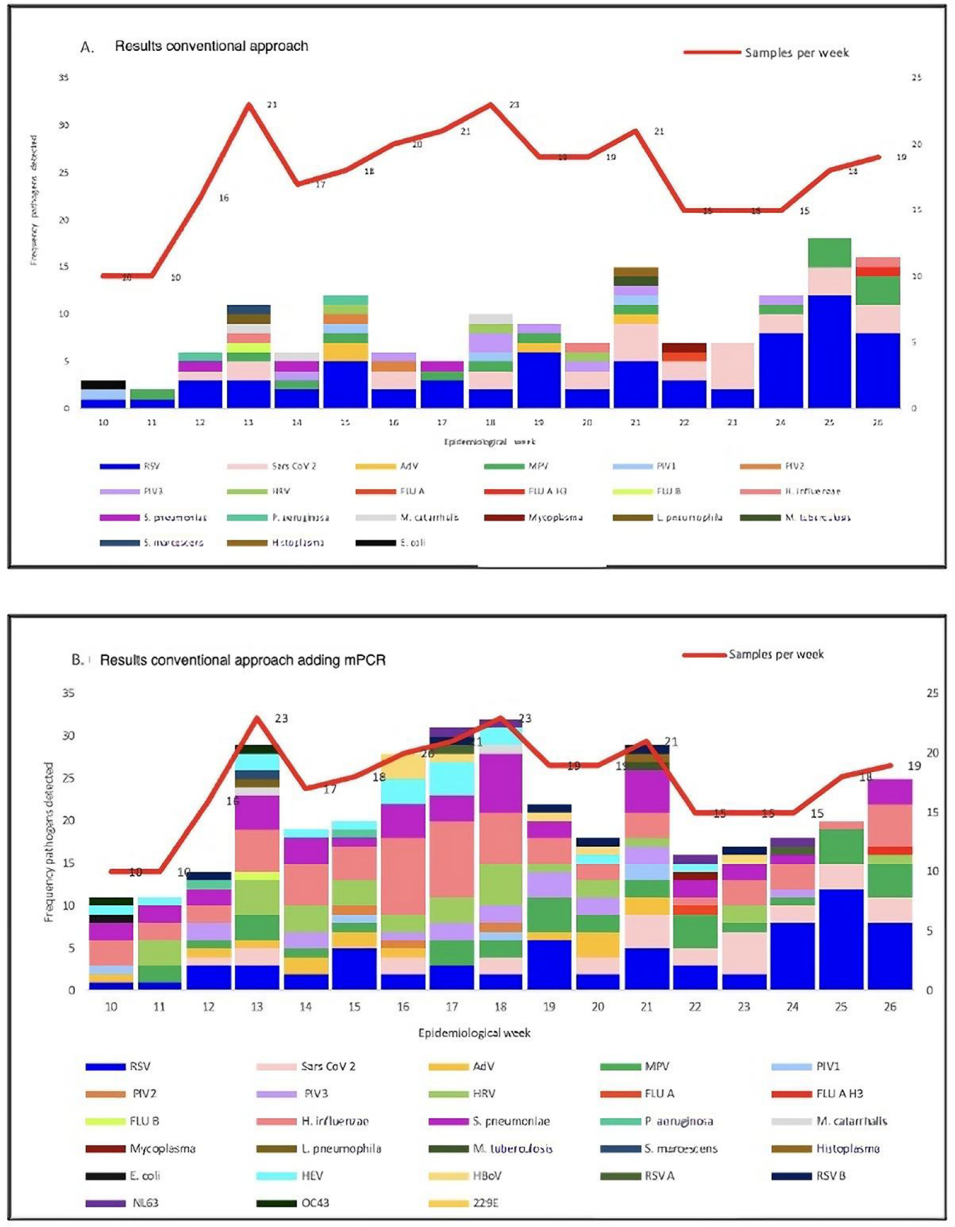
Comparison tracking ARI surveillance from epidemiologic weeks 10–26 (March 6, 2022–June 2, 2022) between the conventional approach **(A)** and conventional approach adding mPCR **(B)** n, number of samples per epidemiological week; RSV, respiratory syncytial virus; RSV A, respiratory syncytial virus A; RSV B, respiratory syncytial virus B; MPV, metapneumovirus; AdV, adenovirus; PIV3, parainfluenza virus 3; PIV1, parainfluenza virus 1; FLU A, influenza A; FLU B, influenza B; HRV, rhinovirus; NL63, coronavirus NL63; OC43, coronavirus OC43; 229E, coronavirus 229E; HEV, enterovirus; HboV, bocavirus.

Using the conventional approach plus mPCR results, the positive samples increased from 131 to 288 (131 by conventional approach plus 157 by mPCR), for a global positive rate of 96.3% (288/299), which means 220% more positive samples, and the number of pathogens identified rose from 24 to 34. Figure 3 demonstrates the broad range of pathogens detected through the addition of mPCR to the conventional surveillance approach.

Following testing with mPCR, 11 samples remained negative, and 10 with sufficient volume were sequenced. The results of sequencing are shown in Supplementary Table S1 (Supplementary Materials) and were submitted to the SRA database (code PRJNA1177867). These results did not identify any new pathogen of concern, outbreaks, or impacted surveillance activities.

Table 1 compares the distribution of sociodemographic features, medical history, clinical manifestations, and antibiotics prescribed among patients testing positive and negative with the conventional approach. In the first group, the median age was one year-old (IQR 0–3 years), while for those with negative samples with the conventional approach, the median age was 2 (IQR 1–5 years). Asthma was the most frequent comorbidity reported in both groups (11.4%, 34/299). Bronchiolitis was the most frequent diagnosis (37.4%) in the group that obtained a result with the conventional approach, while pneumonia was the most frequent diagnosis (35.1%) in the group with negative samples. There were no differences in reported symptoms at admission among groups (Table 1). Two deaths occurred among the enrolled patients, both belonging to the group of patients with positive samples via the conventional approach.

**Table 1:** Clinical, sociodemographic, and clinical characteristics of patients whose samples were positive with the conventional approach and the group with negative samples after the conventional approach.

Characteristic	Positive with the conventional approach (*N* = 131)^a^	Negative samples after the conventional approach (*N* = 168)^a^	Overall (*N* = 299)^a^
Sex			
F	62 (47.3%)	71 (42.3%)	133 (44.5%)
M	69 (52.7%)	97 (57.7%)	166 (55.5%)
Age (years)			
Median (IQR)	1.0 (0.0, 3.0)	2.0 (1.0, 5.0)	2.0 (0.0, 4.0)
Range	0.0, 62.0	0.0, 86.0	0.0, 86.0
Age group (years)			
<1	47 (35.9%)	29 (17.3%)	76 (25.4%)
1	30 (22.9%)	24 (14.3%)	54 (18.1%)
2–4	39 (29.8%)	69 (41.1%)	108 (36.1%)
5–19	10 (7.6%)	31 (18.5%)	41 (13.7%)
20–59	3 (2.3%)	7 (4.2%)	10 (3.4%)
>60	2 (1.5%)	8 (4.8%)	10 (3.3%)
Medical history			
Asthma	13 (9.9%)	21 (12.5%)	34 (11.4%)
Hypertension	2 (1.5%)	8 (4.8%)	10 (3.3%)
COPD	0 (0.0%)	8 (4.8%)	8 (2.7%)
Heart disease	4 (3.1%)	5 (3.0%)	9 (3.0%)
Cancer	5 (3.8%)	4 (2.4%)	9 (3.0%)
Kidney disease	0 (0.0%)	4 (2.4%)	4 (1.3%)
Diabetes mellitus	2 (1.5%)	3 (1.8%)	5 (1.7%)
Obesity	0 (0.0%)	3 (1.8%)	3 (1.0%)
Smoking	2 (1.5%)	2 (1.2%)	4 (1.3%)
Symptoms			
Fever	131 (100.0%)	167 (99.4%)	298 (99.7%)
Cough	130 (99.2%)	166 (98.8%)	296 (99.0%)
Rhinorrhea	87 (66.4%)	113 (67.3%)	200 (66.9%)
Shortness breath	61 (46.6%)	76 (45.2%)	137 (45.8%)
Headache	8 (6.1%)	14 (8.3%)	22 (7.4%)
Diarrhea	14 (10.7%)	14 (8.3%)	28 (9.4%)
Sore throat	4 (3.1%)	9 (5.4%)	13 (4.3%)
Conjunctivitis	2 (1.5%)	3 (1.8%)	5 (1.7%)
Clinical diagnosis			
Pneumonia	33 (25.2%)	59 (35.1%)	92 (30.8%)
Bronchiolitis	49 (37.4%)	27 (16.1%)	76 (25.4%)
Asthmatic crisis	25 (19.1%)	43 (25.6%)	68 (22.7%)
Rhinopharyngitis	22 (16.8%)	27 (16.1%)	49 (16.4%)
COPD exacerbation	0 (0.0%)	5 (3.0%)	5 (1.7%)
Sinusitis	1 (0.8%)	5 (3.0%)	6 (2.0%)
Tracheitis	1 (0.8%)	2 (1.2%)	3 (1.0%)
Antibiotics	47 (35.9%)	73 (43.7%)	120 (40.3%)

^a^ Median (IQR) or frequency (%).

Antimicrobial agents were prescribed to 35.9% (47/131) of the patients with positive samples with the conventional approach, with a slightly higher percentage (43.4%, 73/168) observed among those with negative results. Only viruses were detected in 152 samples (113 samples by the conventional approach and 39 by mPCR), of those 27.6% (42/152) received antibiotics. On the other hand, only bacteria were isolated from 19 samples (10 samples by conventional approach and 9 by mPCR), of which 5 did not receive antibiotics. Finally, virus-bacteria co-infection was detected in 115 samples (5 samples by conventional approach and 107 by mPCR), of those 50.4% (58/115) did not receive antibiotics.

**Figure 4:**
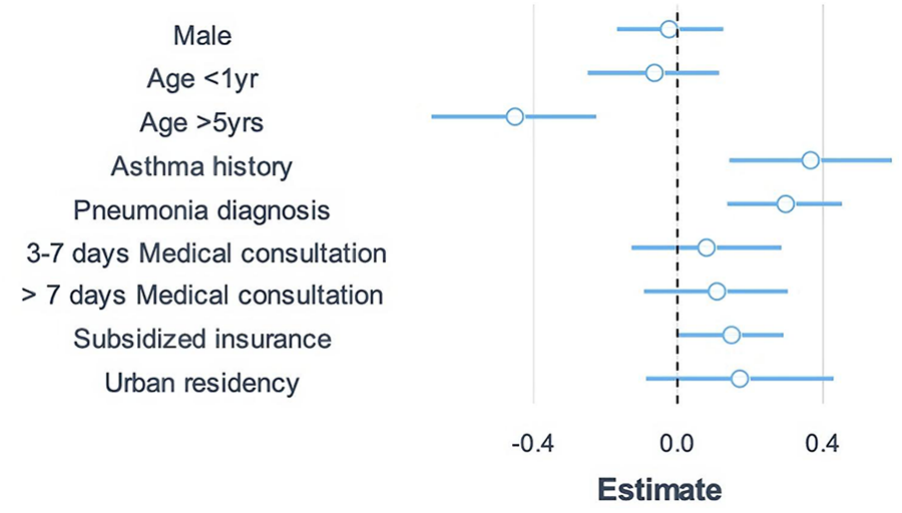
Factors related to a higher number of microorganisms detected in respiratory samples of patients with acute respiratory infection.

A covariate-adjusted Poisson model (Figure 4) showed children bigger than 5 years of age were less likely to have multiple pathogens detected per sample compared with children between 2 and 4 years [rate ratio (RR) 0.64, 95% confidence interval (CI) 0.5–0.8]. Variables associated with a higher number of pathogens per sample included history of asthma compared to other co-morbidities (RR 1.44, 95% CI 1.15–1.8), a diagnosis of pneumonia (RR 1.34, 95% CI 1.58–3.61), and having public health insurance compared with those with payment insurance (RR 1.16, 95% CI 1.00–1.34). No significant differences were observed between males and females (RR 0.97, 95% CI 0.84–1.13), age less than 1 year vs. among age groups (RR 0.93, 95% CI 0.78–1.12), more than 7 days elapsed from symptoms onset vs. less than 6 days (RR 1.02, 95% CI 0.99–1.05), and residing in an urban vs. rural area (RR 1.19, 95% CI 0.92–1.31).

In a hierarchical Poisson regression model, using an approach including mPCR was more likely to result in the detection of multiple pathogens per sample compared with the conventional approach after adjusting by age, sex, history of asthma, and diagnosis of pneumonia (IRR 4.22 95% IC 4.22–5.85) (Supplementary Figure S2).

## 4 Discussion

Previous studies have demonstrated the benefits of mPCR in the clinical management of ILI and SARI (10, 12, 13); however, the utility of mPCR for respiratory pathogen surveillance is less clear. Our study highlights the strengths of mPCR panels for the surveillance of the pathogens associated with ILI and SARI. The current surveillance system in Antioquia, Colombia, which relies mainly on DIF, failed to identify pathogens in a significant proportion of samples, leaving the program at risk of being unable to identify trends and possibly identify outbreaks accurately (14). While the conventional approach used during the study was reasonably robust in identifying viral pathogens, the identification of bacterial pathogens such as *H. influenzae* and *S. pneumoniae,* was poor*,* highlighting important gaps in monitoring outbreaks, antibiotics stewardship, and vaccine efficacy (15).

By implementing mPCR testing to supplement the conventional approach, the number of undiagnosed samples was reduced from 56.2% to 3.6%, greatly enhancing the understanding of the circulating pathogens causing ILI and SARI in the community. In our study, the most frequent pathogens identified by mPCR testing were H. influenza, *S. pneumoniae*, and Rhinovirus. These pathogens are currently not included in the conventional ARI surveillance panel employed in Colombia and many other countries.

Our study, in line with previous research, found that molecular testing led to increased detection of viruses and co-infections of viral and bacterial agents. The study emphasizes the importance of ongoing surveillance of bacteria as contributors to ARI. Including bacterial testing in ARI surveillance, especially for *S. pneumoniae*, is significant as it is currently recognized as the leading cause of mortality in children under 5 years old (16). The high proportion of *S. pneumoniae* and *H. influenzae* detected by mPCR in this study is an interesting finding, future studies should analyze the implication of these bacteria as a pathogen or commensals, and the implication for vaccine strategies. Routine use of mPCR should be considered for ARI surveillance to better monitor the circulation of infectious pathogens in countries not currently employing such methods.

Our study has revealed a noteworthy correlation between having a clinical diagnosis of pneumonia and/or a history of asthma with a higher number of microorganisms detected per sample. This finding might be attributed to the high sensitivity of the mPCR (10); however, some hypotheses include the role of steroid use and the dysregulation in airway immunity in asthma, which may be a risk factor for higher pathogen burden and should be considered in future studies.

Although we utilized both the conventional approach and mPCR testing sequentially, there were still 11 patients in whom no microorganisms were detected. Previous research has investigated the use of sequencing to analyze these types of samples to enhance surveillance efforts and identify novel pathogens that may result in outbreaks (17, 18). However, due to the high cost and sensibility of mPCR testing, our study suggests that incorporating sequencing into a routine ARI surveillance program is unnecessary at this time, particularly in resource-limited settings. However, in situations where there is an abnormally high proportion of mPCR-negative samples and a spike in ILI and SARI, metagenomic sequencing approaches should be employed to detect novel pathogens that may have pandemic potential (19).

Although many countries may lack the resources to routinely employ molecular surveillance of ARI, our study illustrates how a strategy of DIF testing followed by mPCR testing of negatives can improve detection rates. The molecular assay offers superior sensitivity compared to DIF (14, 20). There is a need to better understand the cost-effectiveness of using mPCR in surveillance programs in LMICs. Our study suggests that selective use of mPCR, taking into consideration clinical factors such as medical history of asthma or clinical diagnosis of pneumonia, could be a way of limiting testing to those that may most benefit from an accurate etiologic diagnosis of their ARI. Further, national public health programs that conduct surveillance should select the most appropriate molecular panel based on regional epidemiology, projected risks, lifestyle, vaccination coverage, contact with animals, and other factors.

Some studies have shown that the use of molecular techniques can impact antibiotic prescription practices compared to DIF (13). In our study, no changes were observed in the prescription of antibiotics between those whose samples tested positive with the conventional approach compared to those who did not, however, the impact of additional data made available by the use of mPCR results was not able to be assessed because physicians did not have access to the mPCR results at the time the patient was being on the medical attention and mPCR results include bacteria invading and commensals.

Our study was subject to several limitations. First, given the limited number of adult patients included in this study, the results cannot be generalized for all age groups. Second, because the study was limited to four months March through June 2022, we were unable to account for differences in seasonality, and the impact of the SARS-SoV-2 pandemic (21). Third, the treating physician was not provided with the molecular test results, limiting the ability to assess the impact of mPCR on antibiotic prescription practices compared to DIF (13). Future studies should investigate the costs associated with using mPCR in surveillance and the impact of these technologies on the early detection of outbreaks and public health decision-making. Finally, testing only negative samples limits our ability to fully characterize all potential etiologic agents in the samples. However, given the constraints on resources, our primary objective was to focus on cases with negative results from conventional testing as a starting point.

To summarize, it is recommended that ARI surveillance programs consider incorporating more sensitive diagnostic techniques, including mPCR, to improve the identification of pathogens responsible for respiratory infections. The use of molecular tests is particularly important for the detection of bacteria that may be difficult to recover in routine bacterial cultures and/or the results may be delayed before they can be used for clinical decision-making. This type of surveillance can be useful for informing vaccine introduction and vaccine performance at the population level, as well as informing antibiotic stewardship efforts. Although the availability and accessibility of molecular techniques may be limited and vary by country, the benefits of informing public health and clinical care are clear and should be prioritized by decision-makers.

## Data Availability

The datasets presented in this study can be found in online repositories. The names of the repository/repositories and accession number(s) can be found in the article/Supplementary Material.
