# Supplementary material for: Impact of molecular diagnostic techniques on the acute respiratory infection sentinel surveillance program, Antioquia, Colombia, 2022
Source: Front Epidemiol. 2025 Jan 10;4:1519378. doi: 10.3389/fepid.2024.1519378 (PMC11757876; doi:10.3389/fepid.2024.1519378)
Supplement: Supplementary file 1 [file Datasheet1.pdf]

Supplementary Material

Figure S1. Geographical reference of patients. A. According to the municipality of origin on the map of Antioquia (Colombia). B. According to the collection site on the map of Antioquia (Colombia)

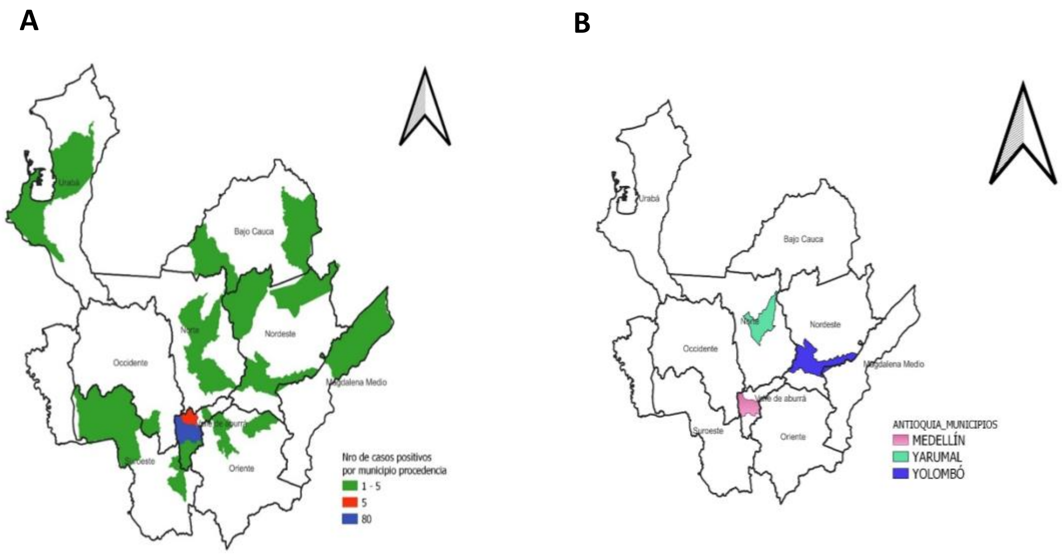


Figure S2. Adjusted weekly trend incidence of microorganisms detected per sample in an approach including mPCR compared with a conventional approach

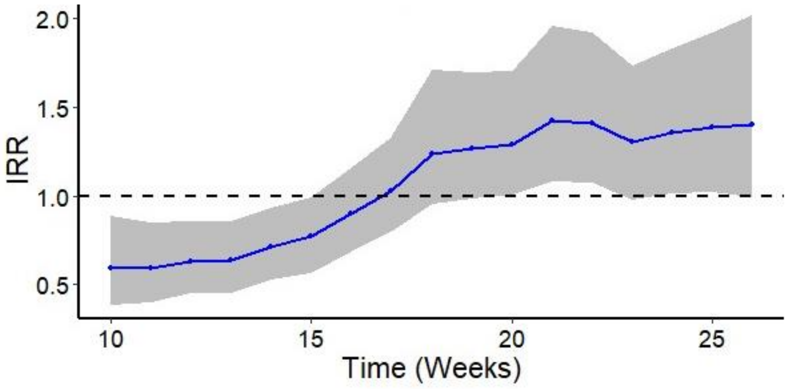

Estimates of the Incidence Rate Radios of the greatest detection of microorganisms per sample

| | IRR Average (95% CI) |
|---|---|
| mPCR | 4.226 (3.05 - 5.849) |
| Age < 1 year | 0.952 (0.661 - 1.372) |
| Age > 5 year | 0.481 (0.327 - 0.703) |
| Male sex | 0.957 (0.716 - 1.280) |
| Asthma history | 1.566 (0.983 - 2.496) |
| Pneumonia diagnosis | 1.583 (1.147 - 2.184) |

Table S1. Summary of sequencing samples results and clinical characteristics (n=10). ***

| **Samples code** | **Total Reads** * | **Microorganism detected**** | **Coverage** | **Depth (X)** | **Clinical Data** |
|---|---|---|---|---|---|
| Resp-007 | 1.153.299 | Dolosigranulum pigrum (B) | 52.0% | 2 | Male, 2y, urban. Rx normal, Bronchiolitis, no antibiotics. |
| Resp-015 | 2.705.229 | Enterovirus D68 (V) | 91.0% | 23 | Male, 10y, urban. Medical history of asthma; Rx air trapping, asthmatic crisis, treated with clarithromycin. |
| Resp-027 | 489.952 | Human rhinovirus C (V) | 19.3% | 0 | Female, 14y, urban. Medical history of univentricular hearts, Fontan procedure; Rx interstitial infiltrates, pneumonia, ceftriaxone, clindamycin. |
| Resp-036 | 466.039 | Rothia mucilaginosa (B) | 30.2% | 0 | Female, 37y, rural, no Rx, Rhinopharyngitis, no antibiotics. |
| | | Scopulariopsis brevicaulis (H) | 17.5% | 0 | |

| | | | | | |
|---|---|---|---|---|---|
| Resp-064 | 1.503.206 | Rothia mucilaginosa (B) | 46.6% | 0 | Male, 86y, rural. Medical history of COPD, DM, HT, CKD; Rx pleural effusion, COPD crisis, clarithromycin, and ampicillin/sulbactam. |
| | | Staphylococcus aureus (B) | 72.1% | 4 | |
| Resp-079 | 9.167.214 | Curvularia geniculata (F) | 20.5% | 0 | Male, 5y, urban, medical history of asthma; No Rx, asthma crisis, no antibiotics. |
| | | Cytomegalovirus (V) | 13.5% | 0 | |
| | | Dolosigranulum pigrum (B) | 53.5% | 1 | |
| Resp-101 | 1.638.955 | Corynebacterium propinquum (B) | 82.4% | 45 | Male, 4y, urban, medical history of brain paralysis and epilepsy; no Rx, diagnosis sinusitis, ampicillin/sulbactam. |
| | | Eikenella corrodens (B) | 27.1% | 0 | |
| | | Moraxella catarrhalis (B) | 51.4% | 2 | |
| | | Streptococcus mitis (B) | 30.4% | 0 | |
| Resp-135 | 9.933.576 | Corynebacterium propinquum (B) | 32.2% | 0 | Female, 85y, urban. Medical history of smoking, COPD, DM, HT, CKD; Rx alveolar infiltrates; diagnosis pneumonia; piperacillin/tazobactam. |
| | | Escherichia coli (B) | 45.9% | 0 | |
| | | Herpes simplex virus 1 (V) | 91.9% | 214 | |
| | | Moraxella catarrhalis (B) | 41.7% | 0 | |
| Resp-164 | 1.749.051 | Fusobacterium nucleatum (B) | 35.7% | 0 | Female, 7y, urban. Medical history of sickle cell anemia. Rx alveolar infiltrates; diagnosis pneumonia; treated with vancomycin, clarithromycin, and ceftriaxone. |
| | | Haemophilus parainfluenzae (B) | 42.6% | 0 | |
| | | Parvimonas micra (B) | 42.8% | 0 | |
| | | Rasamsonia aegroticola (H) | 11.6% | 0 | |
| | | Rhizopus oryzae (Rhizopus arrhizus) (F) | 12.9% | 0 | |
| | | Staphylococcus aureus (B) | 71.1% | 22 | |
| Resp-217 | 2.239.924 | Actinomyces graevenitzii (B) | 54.8% | 1 | Male, 3m, urban. Medical history of heart disease. Rx normal. Dx rhinopharyngitis. No antibiotics. |
| | | Corynebacterium propinquum (B) | 89.3% | 29 | |
| | | Cytomegalovirus (V) | 100.0% | 694 | |
| | | Dolosigranulum pigrum (B) | 39.7% | 0 | |
| | | Moraxella catarrhalis (B) | 100.0% | 520 | |

| | | | | | |
|---|---|---|---|---|---|
| | | Prevotella melaninogenica (B) | 44.5% | 0 | |
| | | Rothia mucilaginosa (B) | 43.4% | 0 | |
| | | Streptococcus mitis (B) | 67.2% | 1 | |

*Total Reads: reads after quality depuration.

** Microorganism detected: (B) Bacteria, (V) Virus, (F) Fungus

*** Accession to cite for these SRA data: PRJNA1177867
